# Supplementary material for: Molecular Alterations in Dog Pheochromocytomas and Paragangliomas
Source: Cancers (Basel). 2019 Apr 30;11(5):607. doi: 10.3390/cancers11050607 (PMC6563419; doi:10.3390/cancers11050607)
Supplement: Supplementary file 1 [file cancers-11-00607-s001.pdf]

Supplementary Materials: Molecular alterations in dog pheochromocytomas and paragangliomas

Esther Korpershoek, Daphne A.E.R. Dieduksman, Guy C.M. Grinwis, Michael J. Day, Claudia E. Reusch, Monika Hilbe, Federico Fracassi, Niels M.G. Krol, André G. Uitterlinden, Annelies de Klein, Bert Eussen, Hans Stoop, Ronald R. de Krijger, Sara Galac and Winand N.M. Dinjens

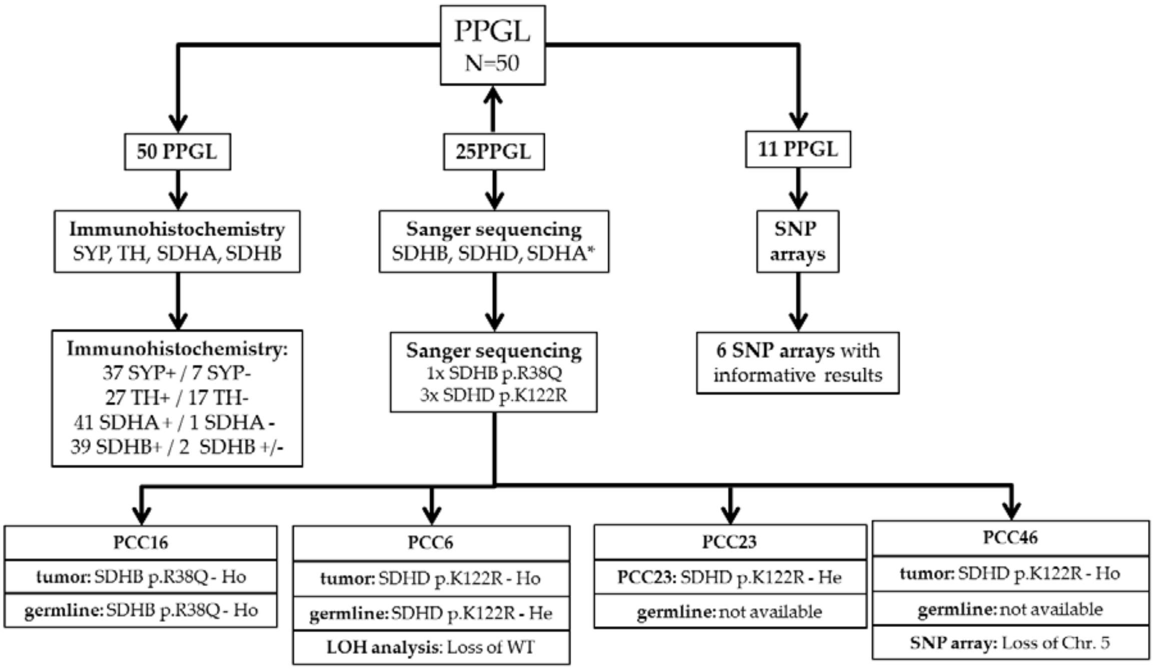

Figure S1. Summary of study design and results. He: heterozygous; Ho: homozygous; WT = wild type allele.

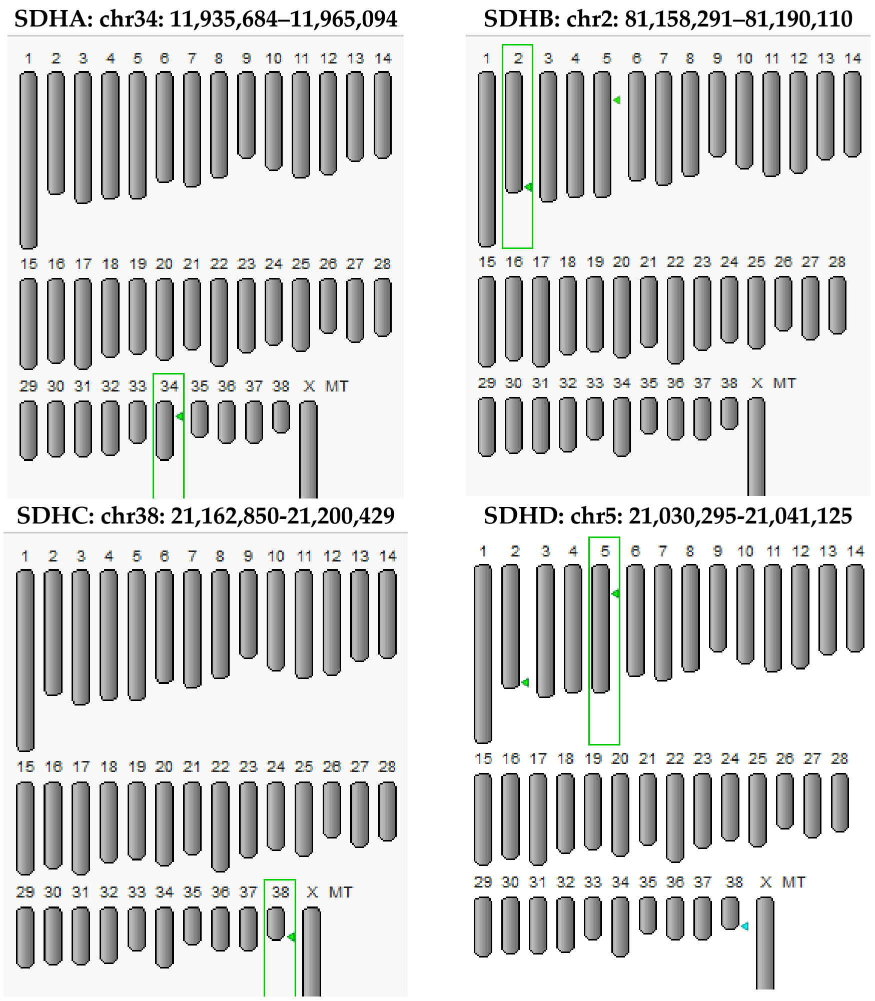

Figure S2. Genomic locations SDH- related genes in the dog genome according NCBI Genome Data viewer. The green box surrounds the chromosome on which the SDHA-D genes are located, with a little green arrow indicating the exact position.

Table S1. Clinical data, immunohistochemistry results and gene mutations in dog PPGL.

Sample no.	Origin	Tumor Location	Side	Dignity	Gender	Breed	Age	Collected during	Tumor material	SYP	TH	SDHB	SDHA	Gene mutation
1	Zurich	PCC	left	B	male	Keeshond	12	surgery	FFPE	pos	pos	het*	neg	WT
2	Zurich	PCC	left	B	female (N)	Standard schnauzer	13	surgery	FFPE	pos	pos	pos	pos	WT
3	Zurich	PCC	right	B	male	Fox terrier	9	surgery	FFPE	pos	pos	pos	pos	WT
4	Zurich	PCC	left	B	female (N)	?	12	necropsy	FFPE	pos	pos	pos	pos	WT
5	Zurich	PCC	left	B	female (N)	Cairn terrier	11	surgery	FFPE	pos	pos	pos	pos	WT
6	Zurich	PCC	right	B	male	Poodle mixed	16	necropsy	FFPE	pos	neg	het*	pos	SDHD
7	Zurich	PCC	left	B	female (N)	Mixed breed	15	necropsy	FFPE	neg	neg	pos	pos	-
8	Zurich	PCC	right	B	female n	Bernese mountain dog	10	surgery	FFPE	pos	pos	pos	pos	-
9	Bologna	PCC	left	B	male	Mixed breed	12	surgery	FFPE	pos	pos	pos	pos	WT
10	Bologna	PCC	left	B	female	Dobermann pinscher	11	necropsy	FFPE	pos	pos	pos	pos	WT
11	Bologna	PCC	right	B	male	Vizsla	14	necropsy	FFPE	pos	pos	pos	pos	WT
12	Bologna	PCC	?	B	female	Mixed breed	7	surgery	FFPE	neg	NA	pos	pos	-
13	Bologna	PCC	?	B	male	Rotweiler	12	necropsy	FFPE	neg	neg	pos	pos	WT
14	Utrecht	PCC	?	B	?	?	?	necropsy	FFPE	NA	pos	NA	NA	WT
15	Utrecht	PCC	left	B	female	Rod Ridgeback	?	necropsy	FFPE	pos	pos	NA	NA	WT
16	Utrecht	PCC	bilateral	B	male	Mixed breed	?	necropsy	FFPE	pos	pos	NA	NA	WT
17	Utrecht	PCC	right	B	male	Dutch shepherd	?	necropsy	FFPE	pos	pos	NA	pos	WT
18	Utrecht	PCC	left	B	male (N)	Golden retriever	8	surgery	FF	NA	NA	pos	pos	WT
19	Utrecht	PCC	right	B	female	Dwergschnautszer	11	surgery	FF	pos	neg	pos	pos	SDHB
20	Utrecht	PCC	right	B	male	Mixed breed	?	?	FF	pos	neg	pos	pos	WT
21	Utrecht	PCC	left	B	female	Rod Ridgeback	9	surgery	FF	pos	pos	pos	pos	WT
22	Utrecht	PCC	left	B	female	Malterzer	11	surgery	FF	pos	pos	pos	pos	WT
23	Utrecht	PCC	left	B	male	Welsh Terrier	10	surgery	FF	pos	pos	pos	pos	SDHD
24	Utrecht	PCC	right	B	male	Dutch shepherd	14	necropsy	FF	NA	NA	NA	NA	-
25	Utrecht	PCC	?	B	female	Malterzer	?	?	FFPE	pos	pos	NA	NA	-

30	Bristol	PGL	aortic	B	male (N)	Boxer	10	necropsy	FFPE	pos	neg	pos	pos	-
31	Bristol	PGL	aoartic	B	male (N)	Border Collie	7	necropsy	FFPE	pos	neg	pos	pos	-
32	Bristol	PGL	aortic	B	female (N)	Mixed breed	8	necropsy	FFPE	neg	neg	pos	pos	-
33	Bristol	PGL	aortic	B	male	Boxer	11	necropsy	FFPE	pos	neg	pos	pos	-
34	Bristol	PGL	aortic	B	male (N)	Bearded Collie	10	necropsy	FFPE	NA	NA	NA	NA	-
35	Bristol	PGL	?	B	female	Mixed breed	11	necropsy	FFPE	pos	pos	pos	pos	-
36	Bristol	PGL	?	B	male (N)	Lurcher	9	necropsy	FFPE	pos	neg	pos	pos	-
37	Bristol	PGL	aortic	B	female (N)	Stafforshire Bull Terrier	11	necropsy	FFPE	pos	neg	pos	pos	-
38	Bristol	PGL	aortic	M	male (N)	Mixed breed	7	necropsy	FFPE	pos	pos	pos	pos	-
39	Bristol	PGL	aortic	B	female (N)	Bull Mastiff	5	necropsy	FFPE	pos	neg	pos	pos	-
40	Bristol	PCC	?	B	?	?	?	?	FFPE	pos	pos	pos	pos	-
41	Bristol	PCC	bilateral	B	male (N)	Cavalier King charles spaniel	12	necropsy	FFPE	pos	pos	pos	pos	-
42	Bristol	PGL	carotid	INV	male (N)	Mixed breed	10	?	FFPE	pos	pos	pos	pos	-
43	Bristol	PGL	aortic	B	male	Labrador	10	necropsy	FFPE	neg	neg	pos	pos	-
44	Bristol	PGL	mediastinum	B	male (N)	Stafforshire Bull Terrier	10	necropsy	FFPE	pos	neg	pos	pos	-
45	Bristol	PCC	right	B	male	Labrador	?	?	FFPE	NA	NA	NA	NA	-
46	Bristol	PGL	?	M	male	German short haired pointer	9	?	FFPE	pos	neg	pos	pos	SDHD
47	Bristol	PGL	mediastinum	B	female (N)	Boxer	10	?	FFPE	pos	pos	pos	pos	-
48	Bristol	PCC	left	INV	female (N)	Whippet	10	?	FFPE	NA	NA	NA	NA	-
49	Bristol	PCC	right	B	male (N)	German Shepherd	4	?	FFPE	pos	neg	pos	pos	-
50	Bristol	PCC	right	M	female (N)	Cavalier King charles spaniel	11	?	FFPE	pos	pos	pos	pos	-
51	Bristol	PCC	?	B	male (N)	Pointer	10	?	FFPE	pos	pos	pos	pos	-
52	Bristol	PGL	mediastinum	INV	male	Vizsla	2	?	FFPE	pos	pos	pos	pos	-
53	Bristol	PGL	aortic	B	male (N)	Jack russel terrier	11	surgery	FFPE	neg	neg	pos	pos	-
54	Bristol	PGL	mediastinum	B	male	Staffordshire Bull Terrier	11	?	FFPE	neg	pos	pos	pos	-

B = benign, M = metastasized, INV = invasive growth, ? = unknown, (N) = neutered, Age is at diagnosis, FFPE = formalin-fixed paraffin-embedded, FF = fresh frozen, pos = immunohistochemically positive, neg = immunohistochemically negative, het* = immunohistochemically heterogeneous, NA = not available. From some dogs FFPE and FF were available from the same tumors (PCC15 = 21, PCC16 = 20, PCC17 = 24, PCC22 = 25).

Table S2. Primers.

Gene	Exon	Forward Primer 5′→3′	Reverse Primer 5′→3′	Ta (C)	Size (bp)
		**Sanger sequencing primers**			
SDHA	1	TCTGCGGACCCTGTGCGC	CGGACCCCCGACATGTCT	61	167
SDHA	1	GTTGGTGGGACAGCGCG	CCCGCGGAGACCGTCCT	61	159
SDHA	2	AAGACACTTAGTGCTCTCCATCTGT	CACCTCTAAGCCAATGATATCACTT	55	175
SDHA	3	TTTGTGCCTCTTTCCGTTGTGC	AAGGCCAAATGCAGCTCGCA	63	170
SDHA	3	CAGTGGTGGTAGGCGCTGGA	CGCCCTGCTGTGCAGGTTC	62	169
SDHA	4	AAGGTTGCTGTCTCTTTGCAG	CAGACCATGCGTGGACACT	55	186
SDHA	5	GAAAGTCTTGATTCCTTCCAGGTGC	ATGGGCCTGCCCGCCTTT	62	156
SDHA	5	TGGACAGAGCCTCAAGTTTGG	GCCTGTCCTAACCAGAGTGTGTCC	57	153
SDHA	6	CACCCTAAATGCAATTGAAACTG	GGACCCATCTTCTATGCACAGTG	57	164
SDHA	6	TGGAAAATGGAGAATGTCGTGG	AGGAAACTGAGACCCAACTTCCC	59	150
SDHA	7	ATGCTGGCATGTCTGTTTTCC	TGAAGGCAATCCCATCCTACC	58	168
SDHA	8	AATTTGCATTTGAAATACAGACCTAGC	AACCTTTCGCCTTGACTGTTAAT	56	152
SDHA	8	GTGGAGAGGGAGGCATTCTC	ATGCATGCATGTGCGCTCT	58	156
SDHA	9	GCCTCACACCATGATGTCCTT	ACATCCACACCGGCAAAGA	57	150
SDHA	9	GTCTGCCTGGCATTTCAGAG	ATTCCTGCCCTATTCCCCAC	57	170
SDHA	10	CAGACGGGTACAAACTGTGC	CCAAAGACGACCAAGTCCAA	55	180
SDHA	10	AACCGCCTCGGAGCCAAC	CAAATCAAGACATGGTAACCTCTCA	54	149
SDHA	11	AATTCTCACTTTTTGTCTGTGTGTTT	AGAGCGTGTGCAGGGCTTA	59	171
SDHA	12	TCCTAGCTGCTTCATGTGAATTA	GCCCCATGGAATGCTCA	55	183
SDHA	13	TCATGAGTACTCGGTGTGTCCCT	CACAGGGGTACGTGGTCAGG	58	182
SDHA	14	TAAACTAATTTCCCTGGCCTCAG	ATCCGTGAGCAGGACACGTT	57	167
SDHA	15	AATATACAAGCGAATACTGTTTTTATGTT	AGTAGGAGCGAATGGCTGGA	56	155
SDHA	15	AGGCTGACTGTGCCACGGTC	CCGGGTAGATGATCTCTATTCCAATG	60	176
SDHB	1	CTAGCGGGTCCCGGGTGGAGG	CCCCGGCCCCGCACCTCAC	70	200
SDHB	2	TGGGTTTGTATCCAATGTGGT	TTATCTCCAGTCTTGTCTGGGTC	55	198
SDHB	2	CGAATCAAGAAATTTGCCATC	TTTTCACATCCTTGGAAGGTTT	55	181
SDHB	3	CAAAGGGCAGGAAGATTGAA	TTTAGGCGCATTTACCCAAG	60	286
SDHB	4	CGAGCCTGGGTACAGAAGC	AGAGAGGGGATGGGAGAGAGAG	57	239
SDHB	5	AATGGGGTGACTGAGAATGC	CTTCTCCCAAGCATCTTTGC	60	253
SDHB	6	CATGCCAGTCTCTTCCATCA	GCCAAAACTAAATCGGATGC	56	249
SDHB	7	GTTGGGTTGCCAGTGCAG	CTCTGACACGTGAGCTCGG	56	230

SDHB	8	AGTTCACTTACCAGTGGTTTCTATTCTC	CTGAAGGGACTCAAGTTGGATG	56	181
SDHD	1	GCTGGGTGACCTTGAGCC	GGATTCCCTGCTTAGAAGC	56	158
SDHD	2	TGTCAGGCCTGTTAAAAGAGAA	CAGATGTAGAGGGCCAGAGC	54	227
SDHD	3	ATGTGTGTTTCCCCCTTTCA	ATGAGACAGGCTCACAGCAA	54	263
SDHD	4	TAATTATTTTTTGCAGTCAAGTTAGCC	TGTACAATCAATTCTGAAGGTATTAAGTC	55	274
			**MICROSATELLITE PRIMERS**		
SDHD	up-stream	AACTACATTTCAGCCAAATTCAACTATTCTG	TCCCGATATCGAGTCCCATATCAG	60	202
SDHD	down-stream	GACTCCACTCCAGCTCTGATG	ACATCATGCAGTCGCTCATTC	56	191

Table S3. Homology overview dog chromosome 5 and 26 with human genome.

Dog Chromosome	Nucleotide Numbers	Human Chromosome	Nucleotide Numbers
5	109084-30009274	11	101451873-135019044
5	30134165-39251262	17	4003704-15723575
5	39060703-42485029	17	16936878-20319299
5	43142020-56072496	1	52825940-67465953
5	56135275-63519382	1	909985-10387998
5	63560328-79803946	16	70075643-90067570
5	79805047-88913024	16	58816246-69943066
26	8469-16869565	12	110050966-133210051
26	17126789-18864828	12	07917843-110049059
26	18976681-25027215	22	24805875-32255978
26	26777542-28762651	22	22341606-24636036
26	31423273-37515643	10	50157832-57800079
26	37523915-38937973	10	87430190-89271465

© 2019 by the authors. Licensee MDPI, Basel, Switzerland. This article is an open access article distributed under the terms and conditions of the Creative Commons Attribution (CC BY) license (http://creativecommons.org/licenses/by/4.0/).
